# Physiological and Transcripts Analyses Reveal the Mechanism by Which Melatonin Alleviates Heat Stress in Chrysanthemum Seedlings

**DOI:** 10.3389/fpls.2021.673236

**Published:** 2021-09-22

**Authors:** Xiaojuan Xing, Yurong Ding, Jinyu Jin, Aiping Song, Sumei Chen, Fadi Chen, Weimin Fang, Jiafu Jiang

**Affiliations:** State Key Laboratory of Crop Genetics and Germplasm Enhancement, Key Laboratory of Landscaping, Ministry of Agriculture and Rural Affairs, College of Horticulture, Nanjing Agricultural University, Nanjing, China

**Keywords:** chrysanthemum, melatonin, high temperature, physiology, RNA-seq

## Abstract

Heat stress limits the growth and development of chrysanthemum seedlings. Although melatonin (MT) has been linked to the heat stress response in plants, research on the underlying molecular mechanisms is scarce. In this study, the regulatory networks of MT on heat stress in chrysanthemum seedlings were explored. Physiological measurements suggested that MT not only reduced malondialdehyde accumulation, hydrogen peroxide content, and superoxide anion free radical generation rate, but also significantly promoted osmotic regulation substance synthesis (proline and soluble protein), antioxidant accumulation (GSH and AsA), and the antioxidant enzyme activities (SOD, POD, CAT, and APX) in chrysanthemum leaves under heat stress. Furthermore, MT increased the fresh weight, dry weight, chlorophyll content, photosynthesis rate, and gas exchange indexes. Further, RNA-seq results revealed 33,497 and 36,740 differentially expressed genes in the S/Con and SMT/ConMT comparisons, respectively. The differences in the comparisons revealed that MT regulated heat shock transcription factors (HSFs) and heat shock proteins (HSPs), and the genes involved in Ca^2+^ signal transduction (*CNGCs* and *CAM/CMLs*), starch and sucrose metabolism (*EDGL, BGLU, SuS*, and *SPS*), hormone (*PP2Cs*, *AUX/IAAs*, *EBFs*, and *MYC2*), chlorophyll metabolism (*HEMA* and *PORA*), flavonoid biosynthesis (*CHS*, *DFR*, and *FNS*), and carotenoid biosynthesis (*DXPS*, *GGDP*, and *PSY*). MT effectively improved chrysanthemum seedling heat-resistance. Our study, thus, provides novel evidence of a gene network regulated by MT under heat stress.

## Introduction

High temperature stress restricts plant growth and development, thereby, severely reducing crop yields (Wilson et al., 2014; Lesk et al., 2016). Heat damage includes leaf curling and yellowing, whole leaf wilting, and leaf edge scorching (Sharma et al., 2016). Moreover, reactive oxygen species (ROS) are produced in excess under heat stress, which in turn causes a series of complex metabolic alterations, including, changes in enzyme activity, in proteins and nucleic acids, and in cell membrane and cytoskeleton stability (Ahammed et al., 2016). Plants have their own antioxidant systems that can effectively scavenge ROS (Baxter et al., 2014). However, excessive accumulation of ROS causes severe disruption of ROS homeostasis, resulting in the oxidation of lipids, DNA, and proteins (Vanderauwera et al., 2011; Baxter et al., 2014). High ambient temperatures enhance transpiration, causing tissue dehydration and even plant death (Locato et al., 2009). Urgent measures are required for the amelioration of heat-induced plant damage; a research area that has received widespread attention (Baninasab and Ghobadi, 2010), particularly, because the plant heat-response system can be overwhelmed by conditions of chronic and severe heat stress.

Acting as an antioxidant and growth regulator (Fleta-Soriano et al., 2017), melatonin was first discovered in the pineal gland of bovines (Arnao and Hernandez-Ruiz, 2014). It plays a vital role in the growth and development of plants, and their resistance to stress (Arnao and Hernandez-Ruiz, 2015), assisting plants to survive and thrive (Reiter et al., 2015). Endogenous regulation and exogenous spraying of melatonin can improve plant resistance to biotic and abiotic stress (Hasan et al., 2015; Shi et al., 2015; Xu et al., 2016). A recent study in *Lolium perenne* revealed that exogenous spraying of melatonin reduced abscisic acid content under heat stress, as well as increased the concentration of endogenous melatonin and cytokinin (Zhang et al., 2017). Heat stress causes misfolding of proteins in plant cells; in tomato, melatonin reduced the ratio of insoluble protein to total protein, thereby protecting plant proteins against heat-induced denaturation (Xu et al., 2016). The application of melatonin increased superoxide dismutase (SOD) (Zhang et al., 2017), and ascorbic acid (AsA)-GSH cycle-related enzyme activities, such as that of ascorbate peroxidase (APX). Melatonin can modulate Ca^2+^ influx through a non-selective Ca^2+^ permeable cation channel (Celik and Nazıroğlu, 2012), and stimulate Ca^2+^ transport across the cellular membranes (Santofimia-Castaño et al., 2014). In kiwifruit seedlings, melatonin can effectively modulate carbon fixation and improve photosynthesis under heat stress by regulating the transcription of triosephosphate isomerase (*TIM*), ribose 5-phosphate isomerase A (*RPI*), and phosphoenolpyruvate carboxykinase (*PCK*) genes (Liang et al., 2019). Melatonin also increase the biosynthesis of polyphenols such as total phenols, flavonoids, and anthocyanins in grape berries (Meng et al., 2019). In addition, melatonin was shown to induce the transcription of heat shock proteins (HSPs) and to promote the degradation of denatured proteins in response to abiotic stress (Shi et al., 2015; Wang et al., 2015; Xu et al., 2016). In plants, melatonin affects root architecture (Pelagio-Flores et al., 2012), organ development (Arnao and Hernandez-Ruiz, 2014), photosynthesis (Arnao and Hernandez-Ruiz, 2015), defense (Weeda et al., 2014), senescence (Byeon et al., 2012; Wang et al., 2013), and stress responses (Kostopoulou et al., 2015; Zhang et al., 2015). Specifically, melatonin effectively maintains photosynthesis in tomato plants growing under heat stress (Ahammed et al., 2018). In maize seedlings, melatonin enhances thermotolerance by modulating antioxidant defense, methylglyoxal detoxification, and osmoregulation systems (Li et al., 2019). In wheat seedlings, melatonin suppressed the heat stress-induced damage by modulating the antioxidant machinery (Buttar et al., 2020). However, whether MT could enhance the thermotolerance of chrysanthemum and the underlying mechanisms is not known.

Chrysanthemum is one of the most widely cultivated cut flowers in the world, thus having high ornamental and economic values. In summer, under heat stress conditions, chrysanthemum seedlings grow slowly, and the leaves curl, turn yellow, and wither. In severe cases, chrysanthemum seedlings can die. If chrysanthemum encounters extreme heat stress during the reproductive growth period, its flowers will die prior to propagation, which will seriously restrict the development of the chrysanthemum industry and the value of ornamental chrysanthemum. Therefore, it is important to identify methods for improving the resistance of chrysanthemum to heat stress conditions. In this study, we explored the melatonin-mediated enhancement of chrysanthemum seedling-stress resistance through its regulation of the physiological and molecular responses involved. Physiologically, our main analyses focused on osmotic regulation substances, peroxides, antioxidant contents, and antioxidant enzymes. Moreover, we highlighted the genes involved in the ROS, the heat shock transcription factor (HSF)-HSP, Ca^2+^ signal transduction, carbon fixation, the starch and sucrose metabolism pathways, hormone signal transduction, and the chlorophyll, flavonoid, and carotenoid metabolic pathways. We elucidated the gene regulatory networks involving melatonin under heat stress; furthermore, our study provides a sound theoretical basis for research on melatonin to improve heat tolerance in plants.

## Materials and Methods

### Plant Materials and Growing Conditions

The chrysanthemum cultivar “Jinba” was obtained from the Chrysanthemum Germplasm Resource Preserving Center (Nanjing Agricultural University, China). Rooted seedlings were transplanted into pots filled with a 1:1 soil/vermiculite mixture and placed in a greenhouse under a 16:8 h light:dark regime; a 25/15°C day/night temperature regime, and 70% relative humidity, until they had formed approximately ten fully expanded functional leaves, excluding young leaves at the same developmental phase. To screen for a suitable melatonin concentration, five concentrations (0, 50, 100, 200, and 400 μM) were tested in a preliminary experiment. The results of preliminary experiments indicated that 200 μM solution was selected as the treatment concentration, owing to it had a stronger effect on the physiological indexes, including growth indicators (i.e., fresh and dry weights), osmotic regulators [i.e., malondialdehyde (MDA), and proline content], and antioxidant enzyme activities [SOD and peroxidase (POD)]. Then chrysanthemum seedlings were uniformly sprayed with 200 μM melatonin every other day for 6 days until the leaves and stems were fully moistened, without the occurrence of dripping. The total volume of melatonin solution sprayed per plant per day was approximately 10 mL with spraying prevention of soils. Control plants (Con) were sprayed with distilled H_2_O (no melatonin). Melatonin was first dissolved in a small amount of alcohol and then formulated to 200 μM melatonin in distilled water. Each treatment was applied to 30 chrysanthemum seedlings and the experiment was performed in triplicates. Subsequently, the chrysanthemum seedlings and controls pre-treated with melatonin or water were subjected to the following treatments: Con, 25°C/15°C with water; stress (S) plants, 40°C/30°C with water; stress with melatonin (SMT), 40°C/30°C with MT, and control with melatonin (ConMT), 25°C/15°C with MT. At 0, 6, 12, 24, and 48 h, leaf samples were collected from the four treatments and stored at −80°C to determine physiological indicators. After 6 days of heat stress, we observed the phenotype of chrysanthemum seedlings pre-treated with melatonin or water, and measured their fresh weight, dry weight, chlorophyll content, photosynthesis rate, and gas exchange indexes.

### Physiological Measurements

Malondialdehyde content was measured by the thiobarbituric acid method after Schmedes and Hølmer (1989). The proline content (Pro) was established by the acid ninhydrin method (Li, 2000), and the Coomassie Brilliant Blue G-250 method was used to determine the content of soluble protein (Bradford, 1976). The soluble sugar content was identified by anthrone colorimetry (Li, 2000), and the hydrogen peroxide (H_2_O_2_) level was determined according to Willekens et al. (1997). The presence of superoxide anion free radicals (O_2_^•–^) was detected using nitroblue tetrazolium staining (Kamrul et al., 2015). Reduced glutathione (GSH) and reduced AsA levels were measured using the method of Ma and Cheng (2003). The activity of SOD was determined based on the method of Giannopolittis and Ries (1997), peroxidase (POD) by the method of Scebba et al. (2001), and catalase (CAT) was determined using the improved method of Kato and Shimizu (1987). APX activity was determined as described by Nakano and Asada (1981). The above measurements were repeated three times, and the average value was used as the representative value for each treatment.

### Measurement of Growth, Photosynthesis Rate and Gas Exchange

Three chrysanthemum seedlings were randomly selected 0 and 6 days after treatment. The seedlings were subsequently cut, rinsed with water, their surface wiped clean with filter paper, and weighed (g), after which, they were oven dried at 105°C for 10 min and then at 80°C to constant weight prior to measurement of dry-mass weight (g). Additionally, three chrysanthemum seedlings were randomly selected, and 0.2 g of fresh-leaf samples were obtained, wiped clean of surface dirt, and then the midrib was removed; the foliar blades were mixed and placed in a test tube, added 10 mL of 95% ethanol and incubated for 12 h in the dark. Absorbance was measured at 665 and 649 nm, and used to calculate chlorophyll a and chlorophyll b, respectively, and total chlorophyll. Rubisco enzyme activity was determined according to the method described by Xia et al. (2009). A portable LI-6800 IRGA (Li-COR, Lincoln, NE, United States) was used to determine net photosynthetic rate (Pn), stomatal conductance (g_*s*_), intercellular CO_2_ concentration (Ci), and transpiration rate (Tr). Three plants were randomly selected for each treatment and mature leaves at plant mid height were selected for the determination of photosynthesis and gas exchange. The open air path and red and blue light sources were selected; quantum flux density was set to 800 μmol⋅m^–2^⋅s^–1^, and the airflow rate in the sample chamber was set to 500 μmol⋅s^–1^; CO_2_ concentration, relative humidity, and temperature were set to 390–410 μmol⋅mol^–1^, 30–40%, and 25°C, respectively.

### RNA Sequencing and Bioinformatics Analysis

According to the measured physiological indicators, we found that the difference between SMT and S was most significant at 24 h after treatment; therefore, we chose 24 h samples for transcriptomic analysis. Total RNA was extracted from Con-24 h, ConMT-24 h, S-24 h, and SMT-24 h using an RNA isolation kit (Waryong, Beijing, China). The leaves collected from five potted plants were considered as one biological replicate, and samples of three such biological replicates were subjected to the DNBSEQ platform for RNA sequencing. Adaptor-polluted, low-quality, and high-content unknown base (N) reads were removed from the raw data (Grabherr et al., 2011). Trinity software (Pertea et al., 2003) was used for *de novo* assembly of clean reads (removed PCR duplication to improve assembly efficiency), and the obtained unigenes were assigned a presumptive function according to homolog deposits in the NR, NT, Swiss-Prot, KEGG, COG, and Pfam databases. The transcriptome datasets are available in the NCBI repository^1^, Accession No. for library PRJNA732569. We identified differentially expressed genes (DEGs) using a false discovery rate (FDR) ≤ 0.05, and | log_2_FoldChange| ≥ 1.0. The heat map of the DEGs was generated using the MeV software.

### Quantitative Real-Time PCR Assay

Eight genes were randomly chosen for quantitative verification to confirm the accuracy of the transcriptome data. Primers used for qRT-PCR were designed using the Primer5 software (Supplementary Table 1). The reference gene *EF-1*α (GenBank: KF305681) was selected as an expression control (Wang et al., 2015). Each sample was represented by three biological replicates and three technical replicates. The specific steps of qRT-PCR were as described previously (Ren et al., 2014). The relative transcriptional expression of DEGs was calculated using the 2^–Δ^ ^Δ^ ^*CT*^ method (Livak and Schmittgen, 2001).

### Statistical Analysis

Using Duncan’s test for data analysis, differences among Con, ConMT, S, and SMT groups were identified as significant at *P* < 0.05. SPSS v17.0 software (SPSS Inc., Chicago, IL, United States) was used for statistical analyses.

## Results

### Physiological Changes of Heat Treatment

**FIGURE 1:**
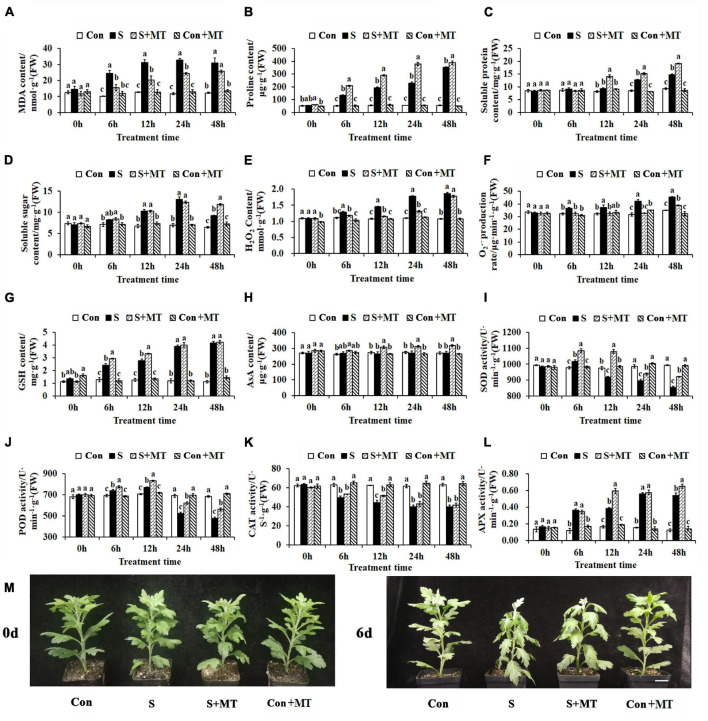
Physiological responses of chrysanthemum seedlings under heat stress. **(A–E)** The content of MDA **(A)**, proline **(B)**, soluble protein **(C)**, soluble sugar **(D)**, and hydrogen peroxide **(E)**. **(F)** The generation rate of superoxide anion free radicals. **(G)** The content of reduced glutathione. **(H)** The ascorbic acid content. **(I–L)** The activities of SOD **(I)**, POD **(J)**, CAT **(K)**, and APX **(L)**. **(M)** Phenotypes after 6 days of heat stress treatment. Con, control; S, heat stress; S + MT, heat stress with exogenous melatonin treatment; Con + MT, control with exogenous melatonin treatment. Error bars indicate SE (*n* = 3). Duncan’s multiple range test was used to analyze significant differences. Different letters indicate significant differences at *P* < 0.05.

To investigate the effects of MT on the resistance of chrysanthemum seedlings to heat stress, the physiological alterations were measured. First, the MDA content was detected, and showed a significant increase in S of 137.5, 144, 178.26, and 152.08% at 6, 12, 24, and 48 h, respectively, and a significant decrease in S + MT of 35.79, 35.25, 25.78, and 17.36% over the same time periods, respectively (Figure 1A). Second, the content of osmotic adjustment substances in chrysanthemum seedlings under heat stress was measured, and compared with control, the proline content of S significantly increased by 154.58, 252.4, 313.31, and 552.32% at 6, 12, 24, and 48 h, respectively. Additionally, the proline content of S + MT increased significantly compared to S by 56.16, 49.88, 65.61, and 10.74% over the same time frames, respectively (Figure 1B). The soluble protein content of S increased by 50.23 and 58.74% compared with control at 24 and 48 h, respectively, whereas that of S + MT began to accumulate at 12 h, increasing by 51.98, 18.17, and 30.1% compared with S at 12, 24, and 48 h, respectively (Figure 1C). The soluble sugar content of S exceeded that of control by 52.22, 87.99, and 42.71% at 12, 24, and 48 h, respectively, while S + MT remained at a higher level than S at 48 h, increasing 29.19% (Figure 1D).

Third, the influence of melatonin treatment on peroxide content in chrysanthemum seedlings under heat stress was investigated. The H_2_O_2_ content of S increased by 14.99, 34.62, 62.35, and 74.16% compared with control at 6, 12, 24, and 48 h, respectively, whereas that of S + MT decreased when compared to S by 8.30, 20.89, 26.60, and 4.57% at the respective time periods (Figure 1E). The generation rate of superoxide anion free radicals of S was significantly increased by 13.55% (6 h), 16.08% (12 h), 33.52% (24 h), and 29.73% (48 h) compared with control, while the generation rate of S + MT was significantly lower than that of S at all times measured and maintained at the control level at 6–24 h (Figure 1F). The antioxidant content of chrysanthemum seedlings under heat stress was measured, and compared with control, the reduced glutathione (GSH) content of S was 87.91, 120.22, 226.19, and 270.89% higher than the control at 6, 12, 24, and 48 h, respectively. Compared with S, the GSH content of S + MT increased by 20.47 and 19.39% at 6 and 12 h, respectively (Figure 1G). The AsA content of S + MT increased when compared to S by 14.46% (12 h), 15.43% (24 h), and 16.91% (48 h) (Figure 1H). Antioxidant enzyme activities were also detected. The SOD activity of S + MT was significantly higher than that of S, which increased by 6.73, 17.41, 4.74, and 7.94% at 6, 12, 24, and 48 h, respectively (Figure 1I). Similarly, POD of S + MT showed greater activity than that of S at all times measured, with increases of 5.06, 7.76, 19.18, and 17.7% compared with S at the four respective times (Figure 1J). The CAT activity of S + MT was increased by 6.65% (6 h), 15.15% (12 h), 7.14% (24 h), and 3.37% (48 h), respectively, compared with S, but there was a significant difference only at 12 h (Figure 1K). Compared with control, the APX activity of S increased by 205.00, 132.14, 261.54, and 333.33% at 6, 12, 24, and 48 h, respectively. At 12 and 48 h, APX was significantly more active in S + MT than S by 53.85 and 19.78%, respectively (Figure 1L). The chrysanthemum seedlings were subjected to heat stress for 6 day; those of Con + MT and CK grew vigorously, followed by S + MT, whereas the S seedlings were the most wilted (Figure 1M). Collectively, these results show that melatonin can effectively alleviate the damage aroused by ROS in chrysanthemum seedlings under heat stress, and is beneficial to the synthesis of osmotic regulation substances and antioxidant contents. Meanwhile, melatonin treatment can effectively reduce the content of MDA and peroxide, and improve the resistance of chrysanthemum seedlings to heat stress.

### Heat-Induced Changes in Growth and Photosynthetic Parameters

**FIGURE 2:**
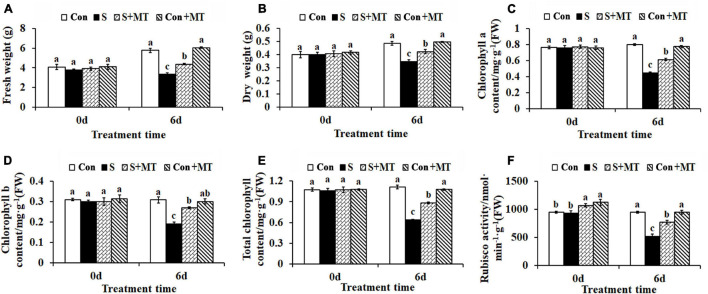
Effects of melatonin on chrysanthemum seedling growth, chlorophyll content and Rubisco enzyme activity under heat stress. **(A)** Fresh weight. **(B)** Dry weight. **(C)** Chlorophyll a. **(D)** Chlorophyll b. **(E)** Total chlorophyll. **(F)** Rubisco activity. Con, control; S, heat stress; S + MT, heat stress with exogenous melatonin treatment; Con + MT, control with exogenous melatonin treatment. Error bars indicate SE (*n* = 3). Duncan’s multiple range test was used to analyze significant differences. Different letters indicate significant differences at *P* < 0.05.

Melatonin treatment alleviated the damage caused by heat stress in chrysanthemum seedlings, whose fresh and dry weights were reduced by 42.27 and 28.77%, respectively, compared with control, after 6 days in the S treatment, while those corresponding to the seedlings under the S + MT treatment were significantly increased by 30.97 and 22.12%, respectively (Figures 2A,B). Similarly, chlorophyll a, chlorophyll b, and total chlorophyll content decreased by 44.12, 38.08, and 42.44%, respectively, in S, compared with the control, while the three increased by 36.79, 40.88, and 38.01%, respectively, under the S + MT treatment, compared to the corresponding values measured under the S treatment (Figures 2C–E). Rubisco is the rate-limiting enzyme in photosynthesis. In this study, Rubisco activity was significantly reduced by 45.28% relative to control after 6 days of heat stress. However, melatonin significantly increased Rubisco activity by 48.28% under S + MT, compared to S (Figure 2F). Additionally, photosynthesis rate (Pn) and gas exchange parameters including, Pn, g_*s*_, Ci, and Tr, all decreased by 42.32, 78.28, 54.79, and 71.45%, respectively, compared with control, while they increased by 29.11, 114.08, 25.0, and 60.32%, respectively, under the S + MT treatment compared with the S treatment (Supplementary Table 2).

### Transcriptome Sequencing and Analysis

To explore the crucial genes and regulatory network at play in chrysanthemum seedlings in response to heat stress and melatonin, we performed transcriptome sequencing on chrysanthemum leaves. Twelve cDNA libraries were sequenced on the DNBSEQ platform, and the total raw reads, total clean reads, and total clean bases obtained were a minimum of 40.48 Mb, 37.58 Mb, and 5.64 Gb (Q20 values > 95.53%, Q30 values > 89.86%, and clean reads > 91.73%), respectively (Supplementary Table 3). After assembly and de-redundancy, 145,639 unigenes were obtained. The total length, average length, N50, N70, N90, and GC content were 174,088,211 bp, 1195 bp, 1667 bp, 1193 bp, 616 bp, and 39.58%, respectively (Supplementary Table 4).

**FIGURE 3:**
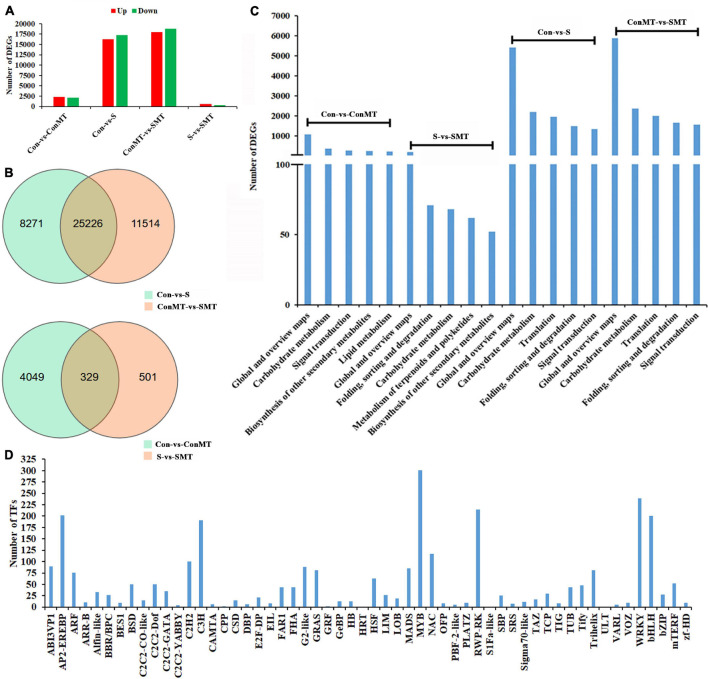
Effects of exogenous melatonin and heat stress on gene expression. **(A)** Number of differentially expressed genes in different comparisons. **(B)** Overlapped differentially expressed genes in different comparisons. **(C)** KEGG analysis in different comparisons (top 5). **(D)** Number of TFs families. Con, control; S, heat stress; SMT, heat stress with exogenous melatonin treatment; ConMT, control with exogenous melatonin treatment.

**FIGURE 4:**
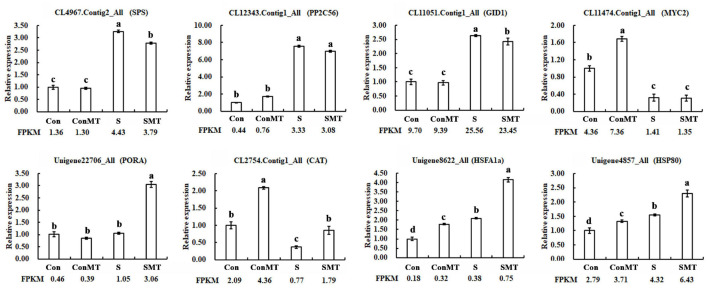
Use of qRT-PCR to verify the accuracy of RNA-Seq. Con, control; S, heat stress; SMT, heat stress with exogenous melatonin treatment; ConMT, control with exogenous melatonin treatment. Error bars represent SE (*n* = 3). Duncan’s multiple range test was used for significant difference analysis, and different letters indicate a significant difference at *P* < 0.05.

The results showed 17,262 downregulated and 16,235 upregulated DEGs in the stress/control (S/Con) comparison; in contrast, in the stress with melatonin/control with melatonin (SMT/ConMT) comparison, downregulated and upregulated DEGs were 18,785 and 17,955, respectively (Figure 3A and Supplementary Table 5). Furthermore, 4378 and 830 DEGs were generated in the comparisons of ConMT/Con and SMT/S, respectively, suggesting that melatonin may control more genes to cope with heat stress. Among the different comparisons, overlapping DEGs were further analyzed. The results showed 329 overlapping DEGs between ConMT/Con and SMT/S, and 25,226 were found between S/Con and SMT/ConMT (Figure 3B). To verify the veracity of our transcriptome data, we randomly selected eight genes for qRT-PCR, and the results proved that RNA-seq was reliable (Figure 4).

In addition, KEGG enrichment analysis was conducted to explore the potential functions of DEGs in response to melatonin and heat stress (Supplementary Table 6). The KEGG pathway in Figure 3C indicates that the DEGs in S/Con and SMT/ConMT were enriched in global and overview maps, carbohydrate metabolism, translation, and signal transduction. In addition, DEGs involved in carbohydrate metabolism and biosynthesis of other secondary metabolites were observed in the ConMT/Con and SMT/S comparisons. Annotation results suggested that highly representative pathways might be indispensable for chrysanthemum survival rate and melatonin regulation under heat stress.

Figure 3D and Supplementary Table 7 list 56 families of TFs. Among them, the number of differentially expressed transcription factors of HSF, MADS, MYB, NAC, TCP, WRKY, and bHLH were 63, 85, 301, 117, 30, 239, and 200, respectively. These results revealed that exogenous melatonin regulated the differential expression of many transcription factors, thus confirming that melatonin plays a vital role in heat stress.

### Genes Involved in the Metabolism of ROS-Scavengers, Heat Shock Transcription Factors and Heat Shock Proteins

**FIGURE 5:**
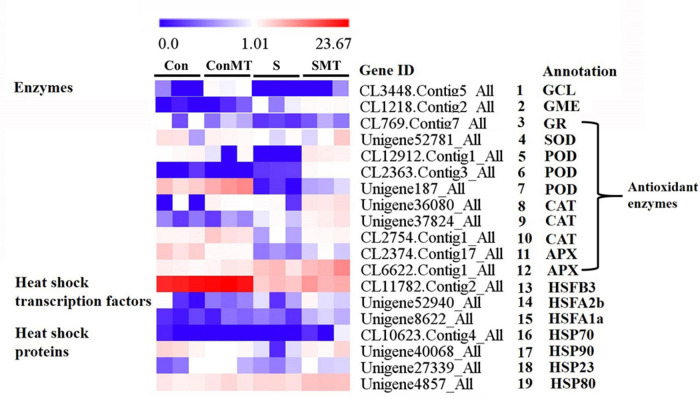
Differentially expressed genes related to enzymes, heat shock transcription factors and heat shock proteins under heat stress in four comparisons. The bar indicates the expression (FPKM) of each gene in Con, ConMT, S, SMT as indicated by blue, white, and red squares. Blue and red represent low and high expression, respectively. Con, control; S, heat stress; SMT, heat stress with exogenous melatonin treatment; ConMT, control with exogenous melatonin treatment. More detailed information is shown in Supplementary Table 8.

Based on the FPKM value, we analyzed the transcriptome data to further clarify the molecular mechanism of exogenous melatonin treatment of chrysanthemum leaves under heat stress conditions, and found 12 enzymes, 3 HSFs, and 4 HSPs related genes (Figure 5 and Supplementary Table 8). The expression of glutamate cysteine ligase (*GCL*) and GDP-D-mannose 3′, 5′-epimerase (*GME*) were significantly more active in SMT than in S (Figure 5, #1 to #2). The DEGs encoding antioxidant enzymes 1 glutathione reductase (*GR*), 1 *SOD*, 3 *POD* (peroxidase), 3 *CAT* (catalase), and 2 *APX* (Figure 5, #3 to #12) respectively, were significantly upregulated in SMT than in S. The expression of the heat-response-related genes *HSFB3*, *HSFA1a*, *HSFA2b*, *HSP23*, *HSP70*, *HSP80*, and *HSP90* was the greatest in the SMT treatment, followed by S (Figure 5, #13 to #19). This shows that spraying chrysanthemum leaves with melatonin under intense heat conditions can effectively improve the stress resistance of the plants.

### Genes Involved in Ca^2+^ Signal Transduction, Carbon Fixation, and Starch and Sucrose Metabolism

**FIGURE 6:**
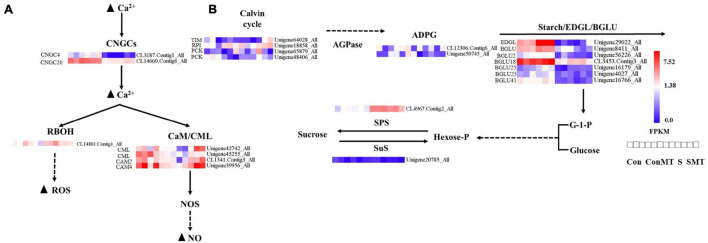
Differentially expressed genes caused by heat stress. **(A)** Calcium signal transduction. **(B)** The metabolism of starch and sugars. The bar represents the expression (FPKM) of each gene in Con, ConMT, S, SMT as indicated by blue, white, and red squares. Blue and red represent low and high expression, respectively. Con, control; S, heat stress; SMT, heat stress with exogenous melatonin treatment; ConMT, control with exogenous melatonin treatment. More detailed information is shown in Supplementary Table 12.

We explored different genes related to calcium signal transduction, photosynthetic biological carbon sequestration, and starch and sucrose metabolism (Figure 6). In S/Con and SMT/ConMT comparisons, heat stress induced *WRKYs* and decreased the expressions of cyclic nucleotide gated channel (*CNGCs*) and mitogen-activated protein kinase kinase kinases (*MAPKKKs*). In S/Con, the expression of respiratory burst oxidase (*RBOH*) was increased, whereas, in SMT/S, it was reduced (Supplementary Table 9). In the SMT/S comparisons, six DEGs involved in the Ca^2+^ signaling pathway were upregulated (Figure 6A). There were four genes in the calmodulin/calmodulin-like protein (CaM/CML) gene family, including two *CML*, one *CAM2*, and one *CAM4*, while two genes (*CNGC4* and *CNGC20*) belonged to the CNGC gene family, and the remaining gene was in the *RBOH* group.

The majority of the genes in the carbon fixation pathway had inhibited expression in relation to heat stress. We emphasized the DEGs in SMT/S. Specifically, the transcription of TIM, PCK, and RPI were upregulated by melatonin (Figure 6B and Supplementary Table 10). Our results (Supplementary Table 11) showed that in ConMT/Con, five genes were induced, including one β-D-*xylosidase* (*BXL*), one *endoglucanase* (*EDGL*), one *trehalose-phosphate phosphatase* (*TPP*), and two *pectinesterase* genes. One sucrose synthase (SuS) encoding gene was reduced, while *polygalacturonase*, *TPP*, and *pectinesterase* were significantly increased in SMT/S. The expression of DEGs encoding ADP-glucose pyrophosphorylase (AGPase), β-glucosidase (BGLU), EDGL, and endoglucane-1, 3-β-glucosidase (EGLC) was reduced under heat stress; conversely, the expression of genes encoding sucrose phosphate synthase (SPS), *TPP*, α-amylase, and β-amylase were induced both in S/Con and in SMT/ConMT comparisons (Supplementary Tables 11, 12).

### Genes Involved in Plant Hormone Signal Transduction

**FIGURE 7:**
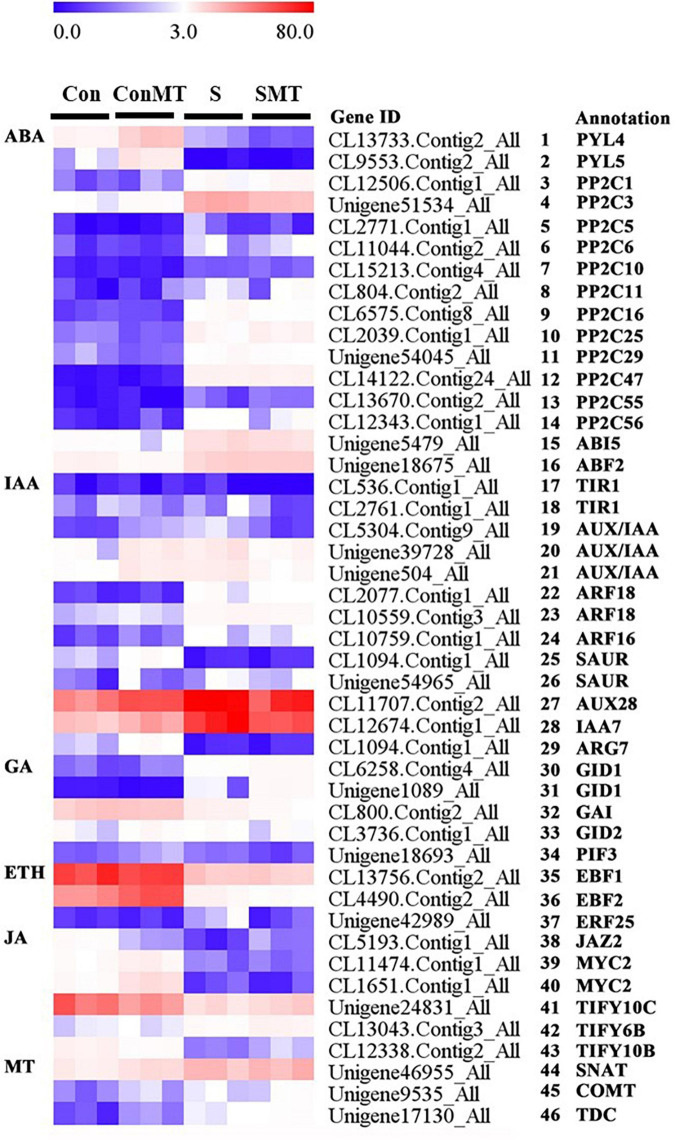
Differentially expressed genes related to plant hormone under heat stress. The bar represents the expression (FPKM) of each gene in Con, ConMT, S, SMT, as indicated by blue, white, and red squares. Blue and red represent low and high expression, respectively. Con, control; S, heat stress; SMT, heat stress with exogenous melatonin treatment; ConMT, control with exogenous melatonin treatment. More detailed information is provided in Supplementary Table 13.

Aiming to clarify the regulatory networks involving melatonin in the plant responses to heat stress, we analyzed the differential expression of genes related to plant-hormone signal transduction (Figure 7 and Supplementary Table 13).

In S/Con and SMT/ConMT, ABA signaling related genes protein phosphatase 2C (PP2C1, PP2C3, PP2C5, PP2C6, PP2C10, PP2C11, PP2C16, PP2C25, PP2C29, PP2C47, PP2C55, and PP2C56) (Figure 7, #3 to #14), the ABA-insensitive (*ABI5*) (Figure 7, #15) gene and *ABF2* (Figure 7, #16) were induced under heat stress, whereas the expression of *PYL4* and *PYL5* (Figure 7, #1 to #2) were inhibited.

Five DEGs including, one *IAA-induced protein* (*ARG7*) (Figure 7, #29), one *AUX28* (Figure 7, #27), one *IAA7* (Figure 7, #28), and two small auxin-up RNA (SAUR) (Figure 7, #25 to #26), were detected in ConMT/Con as induced and related to auxin signal transduction. Heat stress-induced auxin response factors (ARFs) and the expression trends of genes encoding SAURS were inconsistent. In SMT/S, the expression of the auxin receptor *transport inhibitor response 1* (*TIR1*) (Figure 7, #17 to #18) decreased significantly, whereas in S/Con, there was no change in the expression of *TIR1*. Therefore, auxin/indole acetic acid protein (AUX/IAAs) (Figure 7, #19 to #21), *ARFs* (*ARF16* and *ARF18*) (Figure 7, #22 to #24), *SAUR*, and the auxin-responsive proteins *IAA7* and *ARG7* were expressed as hubs in S/Con, whereas in the SMT/ConMT comparison, *TIR1*, *AUX/IAAs*, *ARF18*, *SAUR*, and *ARG7* were pivotal genes.

In terms of GA signaling, heat stress induced the GA receptor (GID1) (Figure 7, #30 to #31), whereas the DELLA proteins (GAI) (Figure 7, #32) were reduced in S/Con and SMT/ConMT, and the expression levels of GID2 (F-box proteins) (Figure 7, #33) and photosensitive interaction factor 3 (PIF3) (Figure 7, #34) were repressed in the latter.

The center gene ethylene-responsive transcription factor 25 (*ERF25*) plays an important role in the heat stress response. In SMT/ConMT and S/Con, EIN3-Binding F-Box protein (*EBF1* and *EBF2*) (Figure 7, #35 to #36) were inhibited. In the SMT/S comparison, *ERF25* was downregulated (Figure 7, #37).

In terms of jasmonic acid signaling, in both S/Con and SMT/ConMT comparisons, heat stress repressed the expression of *TIFY10C* (Figure 7, #41) and *MYC2* (Figure 7, #39 to #40) but there was a similar upregulation of *TIFY6B* (Figure 7, #42) in the latter comparison, whereas *TIFY10B* (Figure 7, #43) and *TIFY10C* were induced in SMT/S.

To reveal how melatonin biosynthesis-related genes were altered in response to heat stress or exogenous melatonin, we analyzed the expression of *SNAT*, *COMT*, and *TDC* genes. In all, ConMT/Con, S/Con, and SMT/S comparisons, *SNAT* (Figure 7, #44), *COMT* (Figure 7, #45), and *TDC* (Figure 7, #46) genes were all upregulated.

### Genes Involved in Chlorophyll, Flavonoid, Carotenoid Metabolism

**FIGURE 8:**
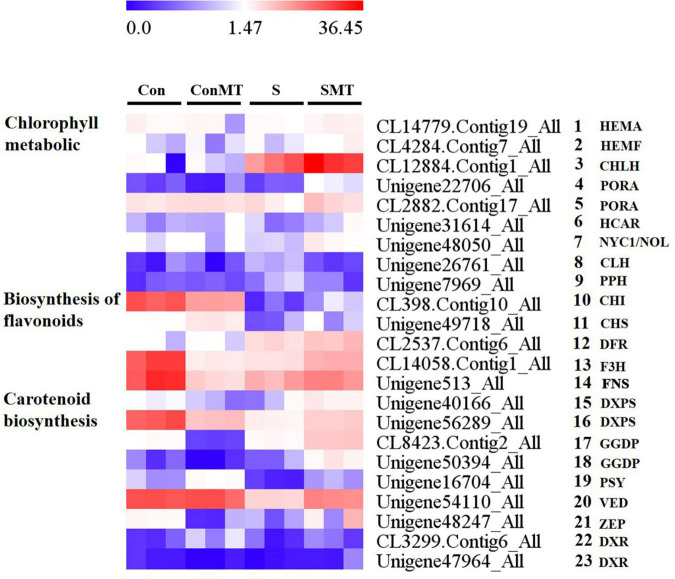
Heat stress modified the expression profiles of chlorophyll, flavonoids, and carotenoid metabolic related genes in four comparisons. The bar represents the expression (FPKM) of each gene in Con, ConMT, S, SMT as indicated by blue, white, and red squares. Blue and red represent low and high expression, respectively. Con, control; S, heat stress; SMT, heat stress with exogenous melatonin treatment; ConMT, control with exogenous melatonin treatment. More detailed information was shown in Supplementary Table 14.

The differential expression of genes involved in chlorophyll, flavonoid, and carotenoid metabolism (Figure 8 and Supplementary Table 14) was identified, which is essential for determining the potential regulatory network of melatonin under heat stress. There were nine differential transcriptions associated with chlorophyll, five genes involved in chlorophyll biosynthesis (Figure 8, #1 to #5), two in the chlorophyll cycle (Figure 8, #6 to #7), and two genes involved in chlorophyll degradation (Figure 8, #8 to #9). The chlorophyll biosynthesis [*HEMA*, *HEMF*, *CHLH*, and protochlorophyllide oxidoreductase (*PORA*)] and chlorophyll cycle (*NYC1/NOL* and *HCAR*) genes had increased abundance in the SMT/S comparison, whereas they remained unchanged in the ConMT/Con comparison. Two transcripts associated with chlorophyll degradation [*CLH* and pheophytinase (*PPH*)] were down-regulated in SMT/S comparison, whereas they were upregulated in the S/Con comparison and remained unchanged in the ConMT/Con comparison. The transcripts associated with flavonoid biosynthesis were (Figure 8, #10 to #14) chalcone isomerase (*CHI*), chalcone synthase (*CHS*), dihydroflavonol 4-reductase (*DFR*), flavanone 3-hydroxylase (*F3H*), and flavone synthase (*FNS*). In SMT/S comparison, these genes were all significantly upregulated, but there was no consistent trend in the other comparisons. In addition, under heat stress conditions, genes related to carotenoid synthesis were affected. In this study, we found nine (Figure 8, #15 to #23) key genes for carotenoid synthesis, two 1-deoxy-D-xylulose-5-phosphate synthase (*DXPS*) (Figure 8, #15 to #16), two geranylgeranyl diphosphate synthase (*GGDP*) (Figure 8, #17 to #18), one phytoene synthase (*PSY*) (Figure 8, #19), one violaxanthin de-epoxidase (*VDE*) (Figure 8, #20), one zeaxanthin epoxidase (*ZEP*) (Figure 8, #21), and two 1-deoxy-D-xylulose-5-phosphate reductoisomerase (*DXR*) (Figure 8, #22 to #23), all of which were significantly upregulated in SMT/S comparisons. In contrast, the expression of *DXPS*, *PSY*, *VDE*, and *ZEP* were significantly downregulated in S/Con comparisons, but there was no significant difference between *GGDP* and *DXR*.

## Discussion

### Effects of Exogenous Melatonin on Osmotic Regulation Substances and Reactive Oxygen Species in Chrysanthemum Seedlings Under Heat Stress

To avoid injury due to heat stress, plants have developed a series of strategies (Chen and Yang, 2019). Thus, for example, H_2_O_2_ plays a critical role in heat stress (Konigshofer et al., 2008; Banti et al., 2010). AtRbohB and AtRbohD are the main synthetases of O_2_^•–^ production by heat stress (Chen and Yang, 2019). In our study, the expression of *RBOH* was significantly upregulated under S, compared to Con treatment; furthermore, we detected that H_2_O_2_ content and O_2_^•–^⋅production rate increased significantly in chrysanthemum seedlings under heat stress. Melatonin can directly remove ROS to stabilize cell membranes and avoid lipid peroxidation under stress (Zhang et al., 2015). Here, the expression of *RBOH*, and H_2_O_2_ and O_2_^•–^⋅levels were reduced in SMT/S. The SOD, POD, CAT, and APX activities of chrysanthemum seedlings enhanced the ability of chrysanthemum seedlings to remove ROS, which is consistent with the results of a related study on lupine (Garnczarska and Bednarski, 2004) and other previous studies (Qi et al., 2018). This could relate to the fact that MT in chrysanthemum heightens antioxidant enzyme activity by increasing the expression of the related genes and reducing the degradation of biological macromolecules, thereby enhancing the ability to remove ROS. When plants are subjected to oxidative stress, GSH is one of the effective scavengers produced by intracellular metabolism and the peroxide processes, which makes plants more resistant to environmental stress, therefore, when plants encounter adversity, the contents of AsA and GSH will change (Yin et al., 2008). Compared with SMT, AsA and GSH contents were lower under S, suggesting that MT enhanced GR activity to accelerate the process of regeneration, and AsA and glutathione synthesized these compounds to relatively high concentrations in chrysanthemum seedlings subjected to heat stress; thereby, improving plant resistance under these conditions.

### Effects of Exogenous Melatonin on Heat Shock Transcription Factor-Heat Shock Protein in Chrysanthemum Seedlings Under Heat Stress

Heat stress increases endogenous melatonin production in Arabidopsis leaves; furthermore, exogenous melatonin enhances the heat resistance of this genus (Shi et al., 2015). Exogenous melatonin and heat stress remarkably induced the expression of A1 heat-shock factors (*HSFA1s*) in *Arabidopsis thaliana*, which are major regulators of the heat stress response (Shi et al., 2015). Studies have shown that exogenous melatonin-enhanced heat resistance was notably attenuated in quadruple knockout *HSFA1* mutants, while *HSFA1*-activated the thermal response gene-transcripts (*HSFA2*, *HSP90*, and *HSP101*), implying they might participate in melatonin-mediated thermotolerance (Shi et al., 2015). HSPs are closely related to heat stress, and *Hsp70* gene overexpression can enhance plant tolerance to this condition (Wang et al., 2004). Consistent with previous reports, the transcription of heat response-related genes (*HSFB3*, *HSFA1a*, *HSFA2b*, *HSP23*, *HSP70*, *HSP80*, and *HSP90*) under SMT was significantly greater than that of S, suggesting that heat-responsive genes played a vital part in melatonin-mediated heat resistance of chrysanthemum leaves.

### Effects of Exogenous Melatonin on Ca^2+^ Signal Transduction in Chrysanthemum Seedlings Under Heat Stress

In plants, calcium signal transduction is the basic mechanism responsible for sensing and responding to environmental stimuli (Duszyn et al., 2019). In signal transduction, CNGCs are involved in the absorption of Ca^2+^ ions as ligand-gated protein channels. A stress-induced Ca^2+^ increase can activate CNGC, leading to a CaM/CML response (Gao et al., 2020). When plants are subjected to heat stress, the specific Ca^2+^ channel on the cell membrane opens, allowing Ca^2+^ to flow along the concentration gradient and activate multiple calcium/calmodulin-binding protein kinases and Ca^2+^-dependent protein kinases, which initiates the expression of downstream genes related to heat stress. In *Ganoderma lucidum*, plasma membrane-mediated extracellular calcium influx, intracellular calcium store release, and other calcium ions from different sources are involved in regulating the increase in intracellular calcium content under heat stress (Zhang et al., 2016), thereby improving the heat tolerance of plants. In our study, in the SMT/S comparisons, *CNGCs* and *CAM/CMLs* were upregulated. Therefore, MT enhanced the stress resistance of plants by activating the expression of *CNGCs* and *CAM/CMLs.*

### Effects of Exogenous Melatonin on Carbon Fixation, and Starch and Sucrose Metabolism in Chrysanthemum Seedlings Under Heat Stress

Heat stress can cause changes in photosynthesis of plants, thereby shortening their life cycle and reducing their productivity (Barnabas et al., 2008). Increasing temperatures inhibit the activities of various enzymes in the Calvin cycle during photosynthesis (Morales et al., 2003). However, by regulating the transcription of *TIM*, *RPI*, and *PCK* genes, MT can effectively modulate carbon fixation and improve photosynthesis under heat stress (Liang et al., 2019). In SMT/S comparison, MT induced the expression of genes encoding *TIM*, *RPI*, and *PCK*.

The high utilization rate of carbohydrates under high-temperature stress is closely related to heat resistance (Roitsch and González, 2004). As sugars act as signaling molecules in the stress response pathway, sucrose and its metabolites can modulate the developmental processes of plants and their response to heat stress through changes in the distribution of carbon, and sugars signaling (Yang X. et al., 2019). Stress induction accelerates the conversion of starch to sugars, thereby, playing a protective role as it contributes to osmotic regulation and a quick energy supply (Dong and Beckles, 2019). When the endosperm develops at high temperatures, starch accumulation decreases (Zhao et al., 2008). SPS plays a vital role in the synthesis of sucrose (Winter and Huber, 2000), our results reveal that the decrease in starch content and induction of α-*amylase*, β-*amylase*, and *SPS* genes under heat stress may promote starch degradability. Exogenous melatonin induced the accumulation of starch and balanced the content of sugars. In line with this hypothesis, exogenous melatonin reportedly improves photosynthesis in plants, which in turn promotes starch accumulation (Sharma et al., 2020). Interestingly, *SuS* was downregulated in the SMT/S comparisons, and *EDGL* was downregulated in S/Con and SMT/ConMT comparisons. Moreover, *BGLU* (*BGLU2*, *BGLU12*, and *BGLU18*) genes were specifically downregulated in the S/Con and SMT/ConMT comparison. Studies have shown that the deficiency of *BGLU18* delays the accumulation of dehydration-induced ABA, indicating that melatonin can stabilize ABA content and abiotic stress responses by regulating BGLU18-mediated ABA-glucose ester hydrolysis (Han et al., 2020). In conclusion, heat stress activated sucrose metabolism and starch degradation, while melatonin enhanced resistance to heat stress by positively regulating the accumulation of carbohydrates and the ratio of starch to sucrose.

### Effects of Exogenous Melatonin on Hormone Signal Transduction in Chrysanthemum Seedlings Under Heat Stress

Studies have shown that heat stress induces plant hormones, such as ABA, AUX, GA, and ETH, which are considered to play a vital role in plant heat tolerance (Kotak et al., 2007). In this study, the differential expression of hormone-related genes was involved in different treatments, further, these genes were related to the increase in heat resistance of chrysanthemum seedlings upon spray treatment with exogenous melatonin under heat stress.

PYR/PYL are ABA receptors that interact with PP2C to reduce the inhibitory effect on SnRK2, thereby regulating the downstream gene ABF (Yang X. et al., 2019; Yang Y. et al., 2019). In the present study, heat stress induced the expression of *PP2Cs*, *ABI5*, and *ABF2*. Studies have shown that overexpression of ABA receptors PYR/PYL might promote ABA signal transduction, thereby increasing plant resistance to abiotic and biotic stress conditions (Liu et al., 2020). Melatonin plays a vital part upstream of the ABA signal (Arnao and Hernandez-Ruiz, 2018), and in this study, melatonin induced the expression level of *PYL4* genes, while the effects of melatonin on the expression of *PP2Cs* did not involve DEGs; therefore, we hypothesized that the MT-mediated expression of *PYL4* enhanced heat stress resistance.

Auxin plays a negative regulatory role in the process of plant resistance to stress, and Aux/IAAs and TIR1/AFB receptors activate a complex regulatory network to regulate the expression of ARF genes (Kazan, 2013; Jung et al., 2015). In accordance with our results, melatonin significantly reduced the transcription of *TIR1* and *Aux/IAA* under heat stress. Studies have found that the IAA content increases under heat stress conditions (Franklin et al., 2011), and in our study, *IAA7* was significantly upregulated by heat stress treatment. In addition, melatonin can regulate the interaction between Aux/IAA and TIR1 to resist heat stress (Naser and Shani, 2016), which was consistent with our study results, suggesting that Aux/IAA multimers significantly inhibited auxin-signal transduction and might improve resistance to heat stress by affecting ROS metabolism. With an increase in GA content, GA interacts with DELLA protein (GAI) after binding to its receptor GID1, thereby causing it to be ubiquitinated and degraded, ultimately activating the GA response (Wang et al., 2021). Under salt stress, melatonin promoted the expression of GA biosynthesis genes *GA20ox* and *GA3ox* in cucumber seedlings, causing the upregulation of GA3 and GA4 (Zhang et al., 2014, 2016). Heat stress decreased GA levels by repressing the expression of GA biosynthetic genes, such as *GA20ox1*, *GA20ox2*, *GA20ox3*, *GA3ox1*, and *GA3ox2* (Toh et al., 2008). Although GA is normally identified as possessing an antagonistic effect on ABA, there is a strong interaction between DELLA proteins and ABF2 (Wang et al., 2020). In accordance with our results, GAI, GID1, GID2, and phytochrome interacting factor 3 were the core components of the GA signal pathway. In studies of tomatoes (Inaba and Chachin, 1988) and apples (Lurie and Klein, 2006), it was found that heat stress (above 38°C) reduced the rate of fruit ripening and the production of ethylene and enhanced respiration. The production of ethylene can improve the resistance of plants to heat stress. Exogenous melatonin slightly increased ethylene generation by inducing the expression of 1-aminocyclopropane-1-carboxylic acid synthase. In contrast, melatonin in etiolated lupine seedlings significantly inhibited ethylene synthesis (Arnao and Hernández-Ruiz, 2007). In the present study, the hub genes *EBF2* and *ERF25* were downregulated by MT under heat stress. *SNAT*, *COMT*, and *TDC* are the melatonin biosynthetic genes (Arnao and Hernandez-Ruiz, 2014). Exogenous melatonin application induced the accumulation of endogenous MT and upregulated the expression of *SNAT* and *COMT* genes in loquat seedlings during a stress period (Wang et al., 2021). In *Agaricus bisporus*, exogenous melatonin application promoted endogenous MT accumulation by increasing the expression levels of *TDC*, *T5H*, *SNAT*, and *ASMT*, which was helpful in protecting membrane integrity (Shekari et al., 2021). Consistent with the previous studies, here, an exogenous melatonin spray promoted the expression of melatonin biosynthesis-related genes *SNAT*, *COMT*, and *TDC* under heat stress.

### Effects of Exogenous Melatonin on Chlorophyll, Flavonoid, and Carotenoid Metabolism in Chrysanthemum Seedlings Under Heat Stress

Heat stress negatively affects various physiological processes of plants, including photosynthesis, and flavonoid and carotenoid metabolism. Heat stress can lead to the degradation of plant chlorophyll, the decrease in photosynthetic rate, the hindrance of photosynthetic electron transfer, and the decrease of enzyme activity related to carbon assimilation (Zhou et al., 2016). Melatonin can improve plant photosynthesis under adversity and improve its resistance to stressors (Biswojit et al., 2018). Consistent with previous results, chlorophyll a, chlorophyll b, and total chlorophyll contents were significantly higher under the SMT than those under the S treatment. The chlorophyll biosynthesis (*HEMA*, *HEMF*, *CHLH*, and *PORA*) and chlorophyll cycle (*NYC1/NOL*) genes were upregulated, and chlorophyll degradation (*CLH* and *PPH*) genes were down-regulated in SMT/S comparison.

Flavonoids are important secondary metabolites in plants. Flavonoids can eliminate various types of ROS, thus resulting in a strong antioxidant capacity (Hernandez et al., 2009). When plants encounter heat stress and other adversity stresses, and a large amount of ROS accumulates in their bodies, flavonoids can degrade this excess ROS and maintain the ROS metabolism balance in plants. Studies have found that melatonin up-regulates the biosynthesis of polyphenols such as total phenols, flavonoids, and anthocyanins in grape berries (Meng et al., 2019). Similar results were found in cabbage, tomato (Sun et al., 2015), and other plants. Melatonin can enhance the activity of phenylalanine ammonia lyase, cinnamic acid-4-hydroxylase, CHS, F3H, leucoanthocyanin reductase, and anthocyanin reductase, and enhance the transcriptional abundance of the corresponding genes, thereby promoting the production of flavonoids, such as anthocyanins in the leaves of kiwifruit, and delaying senescence (Liang et al., 2018). In our study, *CHI*, *CHS*, *DFR*, *F3H*, and *FNS* were all significantly upregulated in the SMT/S comparison, suggesting that exogenous melatonin can improve the stress resistance of chrysanthemum under heat stress conditions.

In addition, studies have found that lutein and some other terpenoids can stabilize and protect the thylakoid membrane from abiotic stress (Camejo et al., 2006). Meanwhile, after overexpressing the chyB gene in *Arabidopsis*, the resistance to heat stress was higher, which indicates that zeaxanthin can prevent oxidative damage of the membrane (Meiri et al., 2010). The survival rate of *Pinctada fucata* decreased with an increase in temperature from 26 to 34°C and with decreasing total carotenoid content. Conversely, a higher total carotenoid content was accompanied by a higher survival rate. This compound, along with and total antioxidant capacity reduced evidently at 30°C with increasing stress (Meng et al., 2016). In our study, the carotenoid synthesis-related genes, such as *DXPS*, *GGDP*, *PSY*, *VDE*, *ZEP*, and *DXR* were significantly upregulated in SMT/S comparisons, and the expression of *DXPS*, *PSY*, *VDE*, and *ZEP* were significantly downregulated in S/Con comparisons, but there was no significant difference between *GGDP* and *DXR*. The results showed that melatonin effectively alleviated the degradation of carotenoids under heat stress conditions and improved the stress resistance of chrysanthemums.

**FIGURE 9:**
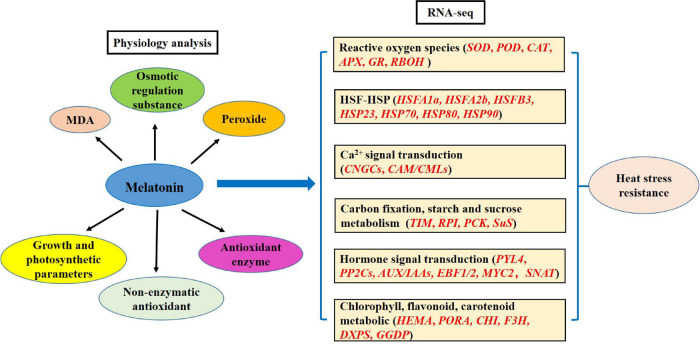
Model of melatonin regulating the adaptability of chrysanthemum seedlings to heat stress.

Overall, exogenous spraying of melatonin improves the resistance of chrysanthemum leaves under heat stress conditions, including physiological and transcription analyses. The physiological aspects mainly include MDA, osmotic regulation substances, peroxides, non-enzymatic antioxidant, and antioxidant enzymes. RNA-seq involves the ROS, HSF-HSP, calcium ion-calmodulin, carbon fixation, starch and sucrose metabolism, hormone, and chlorophyll, flavonoid, and carotenoid pathway-related genes. Based on the above results, we propose the model of the MT-regulated adaptive response to high temperature stress in chrysanthemum leaves (Figure 9).

## Data Availability Statement

The original contributions presented in the study are included in the article/Supplementary Material, further inquiries can be directed to the corresponding author/s.

## Author Contributions

WF, JfJ, and FC conceived and designed the project. XX and YD collected the materials. YD and JyJ carried out the lab work and measured the morphological traits. XX and AS performed the analysis. XX wrote the manuscript with the help from JfJ. FC and SC supervised the experiment. All authors read and approved the final version of the manuscript.

## Conflict of Interest

The authors declare that the research was conducted in the absence of any commercial or financial relationships that could be construed as a potential conflict of interest.

## Publisher’s Note

All claims expressed in this article are solely those of the authors and do not necessarily represent those of their affiliated organizations, or those of the publisher, the editors and the reviewers. Any product that may be evaluated in this article, or claim that may be made by its manufacturer, is not guaranteed or endorsed by the publisher.

## Footnotes

1
https://submit.ncbi.nlm.nih.gov/subs/sra/

## Supplementary Material

Supplementary Table 1 Primer sequences of transcriptome qRT-PCR.

Supplementary Table 2 Effects of melatonin treatment on photosynthesis and gas exchange parameters in chrysanthemum seedlings.

Supplementary Table 3 Summary of sequencing reads after filtering in chrysanthemum leaves were treated with exogenous melatonin.

Supplementary Table 4 Quality metrics of unigenes in chrysanthemum leaves were treated with exogenous melatonin.

Supplementary Table 5 Differentially expressed genes in Con-vs.-ConMT, Con-vs.-S, ConMT-vs.-SMT, and S-vs.-SMT comparisons.

Supplementary Table 6 KEGG analysis (top 5) in Con-vs.-ConMT, Con-vs.-S, ConMT-vs.-SMT, and S-vs.-SMT comparisons.

Supplementary Table 7 Number of TFs families.

Supplementary Table 8 Detailed information of the changes in the expression of enzymes, heat shock transcription factors, and heat shock proteins related genes in Con, ConMT, S, and SMT comparisons.

Supplementary Table 9 Detailed information of the changes in the expression of WRKY, Ca^2+^ signal transduction, and MAPKKKs in Con, ConMT, S, and SMT comparisons.

Supplementary Table 10 Detailed information of the changes in the expression of calvin cycle in Con, ConMT, S, and SMT comparisons.

Supplementary Table 11 Detailed information of the changes in the expression of starch and sucrose in Con, ConMT, S, and SMT comparisons.

Supplementary Table 12 Detailed information of the changes in the expression of the vital genes in the Ca^2+^ signal transduction, starch and sugars metabolism in Con, ConMT, S, and SMT comparisons.

Supplementary Table 13 Detailed information of the changes in the expression of hormone related genes in Con, ConMT, S, and SMT comparisons.

Supplementary Table 14 Detailed information of the changes in the expression of chlorophyll, flavonoids, and carotenoid metabolic related genes in Con, ConMT, S, and SMT comparisons.

## Abbreviations

ABI5: ABA-insensitive
AGPase: ADP-glucose pyrophosphorylase
ARF: auxin response factor
ARG7: IAA-induced protein
APX: ascorbate peroxidase
AsA: ascorbic acid
AUX/IAAs: auxin/indole acetic acid protein
BGLU: βglucosidase
BXL: βD-xylosidase
CAT: catalase
CAM/CMLs: calmodulin/calmodulin-like protein
CHI: chalcone isomerase
CHLH: magnesium-chelatase subunit ChlH
CHS: chalcone synthase 2
Con: Control
ConMT: control with melatonin
CLH: hydroxymethyl chlorophyll a reductase
CNGCs: cyclic nucleotide gated channel
DEGs: differentially expressed genes
DFR: dihydroflavonol 4-reductase
DXPS: 1-deoxy-D-xylulose-5-phosphate synthase
DXR: 1-deoxy-D-xylulose-5-phosphate reductoisomerase
EBFs: EIN3-Binding F-Box protein
EDGL: endoglucanase
EGLC, endoglucane-1: 3- βglucosidase
ERF25: ethylene-responsive transcription factor 25
FDR: false discovery rate
FNS: flavone synthase
F3H: flavanone 3-hydroxylase
GCL: glutamate cysteine ligase
GGDP: geranylgeranyl diphosphate synthase
GME: GDP-D-mannose 3′, 5′epimerase
GR: glutathione reductase
GSH: glutathione
HEMA: glutamyl-tRNA reductase
HEMF: coproporphyrinogen III oxidase
H_2_O_2_: hydrogen peroxide
HSFs: heat shock transcription factors
HSPs: heat shock proteins
MAPKKKs: mitogen-activated protein kinase kinase kinases
MDA: malondialdehyde
MT: melatonin
NYC1/NOL: chlorophyllide a oxygenase
O_2_^•–^: superoxide anion free radicals
PCK: phosphoenolpyruvate carboxykinase
PIF3: photosensitive interaction factor 3
POD: peroxidase
PORA: protochlorophyllide oxidoreductase
PP2Cs: protein phosphatase 2C
PPH: pheophytinase
Pro: proline
PSY: phytoene synthase
RBOH: respiratory burst oxidase
ROS: reactive oxygen species
RPI: ribose 5-phosphate isomerase A
S: stress
SAUR: small auxin-up RNA
SOD: superoxide dismutase
S/Con: stress/control
SMT: stress with melatonin
SMT/ConMT: stress with melatonin/control with melatonin
SPS: sucrose phosphate synthase
SuS: sucrose synthase
TIM: triosephosphate isomerase
TIR1: transport inhibitor response 1
TPP: trehalose-phosphate phosphatase
VDE: violaxanthin de-epoxidase
ZEP: zeaxanthin epoxidase.
