# Supplementary material for: Physiological and Transcripts Analyses Reveal the Mechanism by Which Melatonin Alleviates Heat Stress in Chrysanthemum Seedlings
Source: Front Plant Sci. 2021 Sep 22;12:673236. doi: 10.3389/fpls.2021.673236 (PMC8493036; doi:10.3389/fpls.2021.673236)
Supplement: Supplementary Table 1 — Primer sequences of transcriptome qRT-PCR. [file Table_1.DOC]

**Supplementary Table S1 |** Primer sequences of transcriptome qRT-PCR.

| Primer name | Sequence（5’-3’） |
| --- | --- |
| CmEF1α-RT-F | TTTTGGTATCTGGTCCTGGAG |
| CmEF1α-RT-R | CCATTCAAGCGACAGACTCA |
| CmCAM4-RT-F | CCAAATGCCACAAGGAGATGA |
| CmCAM4-RT-R | GTGACTTGGCTTGAGGACGAG |
| CmSPS-RT-F | GGGACAGAAGATGGCTATGGTT |
| CmSPS-RT-R | TGAATCCGCATACTTAGGTGGT |
| CmPP2C56-RT-F | ATCAGTGGATTCTAACCCGACC |
| CmPP2C56-RT-R | GACCCGATTCAAAGAAATGGAT |
| CmGID1-RT-F | TTTCCCAACTTTCGTCATCACT |
| CmGID1-RT-R | CTCCTTTCACTAATCTCAGCCAG |
| CmMYC2-RT-F | ATTGTCACCGTAAAGGCAGCA |
| CmMYC2-RT-R | AGGTTTTTGGAGACGGGGAG |
| CmPORA-RT-F | GTTCGCATTCGTTTCGTTTTC |
| CmPORA-RT-R | TGGTTCTGTTGGCTTCTTGGTA |
| CmPOD-RT-F | TTGACTGGAACACGAGAATGAAA |
| CmPOD-RT-R | ATAGGTCCCCATCACTCTCGTT |
| CmCAT-RT-F | GTCATTGGTGTGGTGCCCTAC |
| CmCAT-RT-R | TGACATCAAGAGGATAAGCGACTG |
| CmHSFA1a-RT-F | CTCGCTGTTTATCCCCATTAGG |
| CmHSFA1a-RT-R | CTGGGTGAAAGCCCGAGAA |
| CmHSP80-RT-F | TTTGATGTCAGAAGGAGGCGT |
| CmHSP80-RT-R | GTTGCCTCGTTTTCTGCCAT |
