# Supplementary material for: Physiological and Transcripts Analyses Reveal the Mechanism by Which Melatonin Alleviates Heat Stress in Chrysanthemum Seedlings
Source: Front Plant Sci. 2021 Sep 22;12:673236. doi: 10.3389/fpls.2021.673236 (PMC8493036; doi:10.3389/fpls.2021.673236)
Supplement: Supplementary Table 2 — Effects of melatonin treatment on photosynthesis and gas exchange parameters in chrysanthemum seedlings. [file Table_2.DOC]

**Supplementary Table 2 |** Effects of melatonin treatment on photosynthesis and gas exchange parameters in chrysanthemum seedlings.

| Treatment | Net photosynthetic rate（Pn, μmol•  m^-2^•s^-1^） | Stomatal conductance（g_s_, mol•m^-2^•s^-1^） | Intercellular CO_2_ concentration（Ci, μmol•mol^-1^） | Transpiration rate（Tr, mmol•m-^2^•s^-1^） |
| --- | --- | --- | --- | --- |
| Con | 14.63±0.62a | 0.149±0.017a | 368.7±19.9a | 3.62±0.08a |
| S | 8.44±0.03c | 0.032±0.005c | 166.7±7.80c | 1.03±0.06c |
| S+MT | 10.90±0.06b | 0.069±0.004b | 208.3±7.20b | 1.65±0.11b |
| Con+MT | 14.20±0.15a | 0.147±0.011a | 392.3±11.7a | 3.76±0.13a |
