# Supplementary material for: Physiological and Transcripts Analyses Reveal the Mechanism by Which Melatonin Alleviates Heat Stress in Chrysanthemum Seedlings
Source: Front Plant Sci. 2021 Sep 22;12:673236. doi: 10.3389/fpls.2021.673236 (PMC8493036; doi:10.3389/fpls.2021.673236)
Supplement: Supplementary Table 3 — Summary of sequencing reads after filtering in chrysanthemum leaves were treated with exogenous melatonin. [file Table_3.DOC]

**Supplementary Table S3 |** Summary of sequencing reads after filtering in chrysanthemum leaves were treated with exogenous melatonin.

| Sample | Total raw reads (Mb) | Total clean reads (Mb) | Total clean bases (Gb) | Q20 percentage (%) | Q30 percentage (%) | Clean reads ratio (%) |
| --- | --- | --- | --- | --- | --- | --- |
| Con1 | 45.57 | 42.44 | 6.37 | 95.75 | 90.29 | 93.12 |
| Con2 | 45.57 | 42.72 | 6.41 | 95.57 | 89.92 | 93.73 |
| Con3 | 41.06 | 38.07 | 5.71 | 95.82 | 90.48 | 92.72 |
| Con1MT | 40.48 | 37.58 | 5.64 | 95.53 | 89.86 | 92.84 |
| Con2MT | 45 | 42 | 6.3 | 95.62 | 90.08 | 93.32 |
| Con3MT | 42.62 | 39.14 | 5.87 | 95.75 | 90.36 | 91.83 |
| S1 | 45.57 | 42.52 | 6.38 | 95.69 | 90.19 | 93.29 |
| S2 | 45.57 | 42.19 | 6.33 | 95.82 | 90.47 | 92.58 |
| S3 | 45.57 | 41.99 | 6.3 | 95.76 | 90.33 | 92.13 |
| S1MT | 45.57 | 42.16 | 6.32 | 95.61 | 90.06 | 92.51 |
| S2MT | 41.97 | 38.5 | 5.77 | 95.6 | 90.06 | 91.73 |
| S3MT | 45.57 | 42.28 | 6.34 | 95.65 | 90.1 | 92.78 |
