# Supplementary material for: Physiological and Transcripts Analyses Reveal the Mechanism by Which Melatonin Alleviates Heat Stress in Chrysanthemum Seedlings
Source: Front Plant Sci. 2021 Sep 22;12:673236. doi: 10.3389/fpls.2021.673236 (PMC8493036; doi:10.3389/fpls.2021.673236)
Supplement: Supplementary Table 4 — Quality metrics of unigenes in chrysanthemum leaves were treated with exogenous melatonin. [file Table_4.DOC]

**Supplementary Table S4 |** Quality metrics of unigenes in chrysanthemum leaves were treated with exogenous melatonin.

| Sample | Total number | Total length | Mean length | N50  bp | N70  bp | N90  bp | GC percentage (%) |
| --- | --- | --- | --- | --- | --- | --- | --- |
| Con1 | 68705 | 55895772 | 813 | 1164 | 748 | 370 | 40.05 |
| Con2 | 65313 | 52523991 | 804 | 1154 | 740 | 362 | 40.12 |
| Con3 | 63342 | 51530872 | 813 | 1176 | 752 | 367 | 40.16 |
| Con1MT | 63043 | 50640138 | 803 | 1149 | 736 | 365 | 40.23 |
| Con2MT | 66924 | 53407728 | 798 | 1140 | 732 | 364 | 40.06 |
| Con3MT | 64821 | 51736737 | 798 | 1140 | 731 | 361 | 40.13 |
| S1 | 70752 | 57565716 | 813 | 1164 | 751 | 370 | 40.08 |
| S2 | 71461 | 58055798 | 812 | 1145 | 749 | 377 | 39.99 |
| S3 | 73921 | 60574875 | 819 | 1153 | 757 | 380 | 39.98 |
| S1MT | 72890 | 58416431 | 801 | 1148 | 737 | 365 | 39.91 |
| S2MT | 67550 | 52966383 | 784 | 1115 | 721 | 359 | 40.11 |
| S3MT | 72667 | 58097734 | 799 | 1123 | 735 | 373 | 39.96 |
| All-Unigene | 145639 | 174088211 | 1195 | 1667 | 1193 | 616 | 39.58 |
